# (2*R*,3*R*)-1,4-Dioxa­spiro­[4.4]nonane-2,3-di­carb­oxy­lic and (2*R*,3*R*)-1,4-dioxa­spiro­[4.5]decane-2,3-di­carb­oxy­lic acids

**DOI:** 10.1107/S2056989018009593

**Published:** 2018-07-06

**Authors:** Mikhail E. Minyaev, Dmitrii M. Roitershtein, Alexey A. Vinogradov, Ivan V. Ananyev, Ilya E. Nifant’ev

**Affiliations:** a A.V. Topchiev Institute of Petrochemical Synthesis, Russian Academy of Sciences, 29 Leninsky Prospect, 119991, Moscow, Russian Federation; b N.D. Zelinsky Institute of Organic Chemistry, Russian Academy of Sciences, 47 Leninsky Prospect, Moscow, 119991, Russian Federation; c A.N. Nesmeyanov Institute of Organoelement Compounds, Russian Academy of Sciences, 28 Vavilova Str., Moscow, 119991, Russian Federation; d Chemistry Department, M.V. Lomonosov Moscow State University, 1 Leninskie Gory Str., Building 3, Moscow, 119991, Russian Federation

**Keywords:** tartric acid, 1,3-dioxolane, ketals, NMR, hydrogen bonding, crystal structure

## Abstract

The title compounds, C_9_H_12_O_6_ and C_10_H_14_O_6_, were formed by careful hydrolysis of the corresponding diethyl esters. Their single crystals were grown from an ethyl acetate/hexane mixture. Crystals of both compounds have monoclinic (*P*2_1_) symmetry with a single mol­ecule in the asymmetric unit. Both crystal structures are very similar and display four –CO—OH⋯O=C(OH)– hydrogen bonds, forming a two-dimensional double-layered framework.

## Abstract 2

The title compounds, (CH_2_)_*n*_C_3_H_2_O(COOH)_2_ (*n* = 4, 5), display inter­molecular hydrogen bonding, forming a two-dimensional framework.

## Chemical context   

Transition-metal catalysis has developed as a powerful tool to create a variety of carbon–carbon and carbon–heteroatom bonds. Enanti­oselective versions of these reactions are especially inter­esting in the light of the possible pharmaceutical applications. The general route to such processes supposes the use of transition metal complexes with chiral ligands (Yang *et al.*, 2017 ▸). Therefore, easily accessible ligands of this type are of great importance for homogenous catalysis. Chiral phosphine ligands and amino acids are the most popular in this respect (Crassous, 2009 ▸). Examples of chiral carboxyl­ate ligands are also known (Saget *et al.*, 2012 ▸), which can be useful in the synthesis of chiral coordination compounds and mat­erials derived from them (Lam *et al.*, 2011 ▸). Various tartaric acid derivatives, which are also used in organic synthesis as chiral auxiliary agents to create chiral building blocks (Kassai *et al.*, 2000 ▸; Seebach *et al.*, 2001 ▸), might be particularly useful in solving the stated problem. Herein we report the synthesis and structures of two tartaric acid derivatives that may potentially be used as synthetic precursors of chiral transition-metal catalysts.
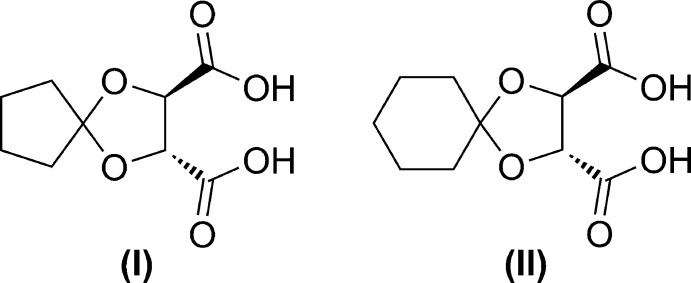


**Figure 1:**
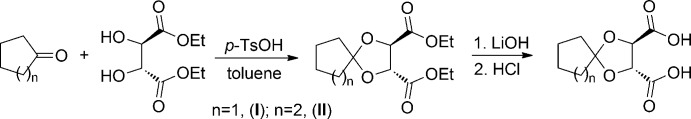
The synthesis of the title compounds (I) and (II).

Condensation of cyclo­penta­none or cyclo­hexa­none with (2*R*,3*R*) diethyl tartrate led to the formation of the corres­ponding ketals, careful hydrolysis of which allowed us to prepare the title acids (Fig. 1 ▸).

## Structural commentary   

**Figure 2:**
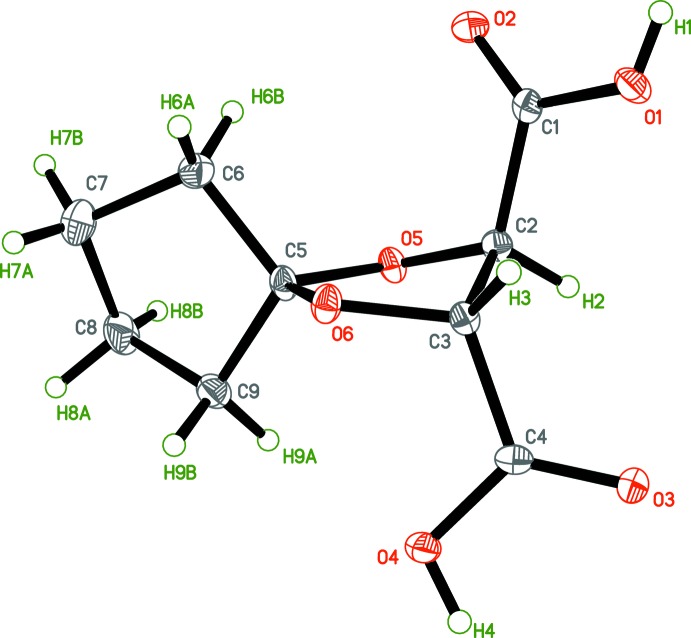
The structure of (2*R*,3*R*)-1,4-dioxa­spiro­[4.4]nonane-2,3-di­carb­oxy­lic acid, (I). Displacement ellipsoids are drawn at the 50% probability level.

**Figure 4:**
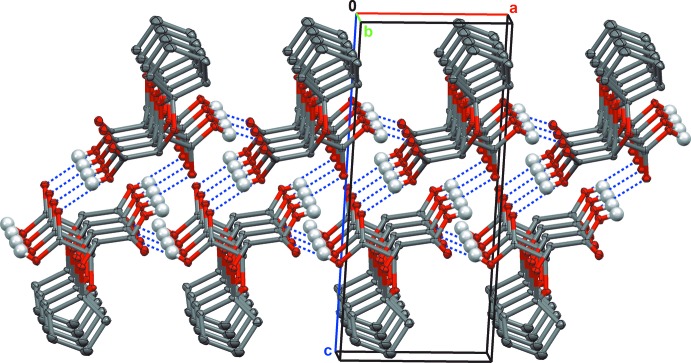
The packing of (I) parallel to (010). Two inter­acting mol­ecular layers are shown. Only the H atoms involved in hydrogen bonding (blue dashed lines) have been included. Displacement ellipsoids are drawn at the 50% probability level.

The structures of tartaric acid derivatives (I) and (II) were found as anti­cipated (Figs. 2 ▸ and 3 ▸, respectively), having a single mol­ecule in the asymmetric unit. The 1,3-dioxolane, cyclo­pentane [in (I)] and cyclo­hexane [in (II)] fragments have the usual conformations. The C—C, C—O and C=O bond lengths are within regular distances (Tables 1 ▸ and 2 ▸). A detailed structural and conformational analysis for the crystal structures of some related acetals *R*′C_3_H_3_O_2_(CO*R*)_2_ (*R* = NH_2_, OAlkyl, OH; substituent *R*′ is at the 2-position of the 1,3-dioxolane ring) was given by Eissmann *et al.* (2012 ▸). Although the absolute structures of (I) and (II) cannot be unambiguously determined using the Flack parameter (Flack, 1983 ▸; Parsons, *et al.*, 2013 ▸) with the *SHELXL* program (Sheldrick, 2015 ▸), the chirality at carbon atoms C2, C3 (2*R*,3*R*) is initially known from their synthetic precursor (diethyl l-tartrate), and has been also confirmed for (2*R*,3*R*)-diethyl 1,4-dioxa­spiro­[4.5]decane-2,3-di­carboxyl­ate, and for (II) by optical rotation measurements (see the experimental section). The mol­ecules of (I) and (II) have very similar positions in the unit cells, making the structures nearly isomorphous, but the *c* axis in (II) is elongated by almost 1.5 Å compared with that in (I) (see Table 5 ▸ below) because of the presence of an additional –(CH_2_)– unit in the cyclo­alkane fragment in (II) (see Fig. 4 ▸ for the alignment of the cyclo­alkane fragments in the unit cell).

## Supra­molecular features   

**Figure 5:**
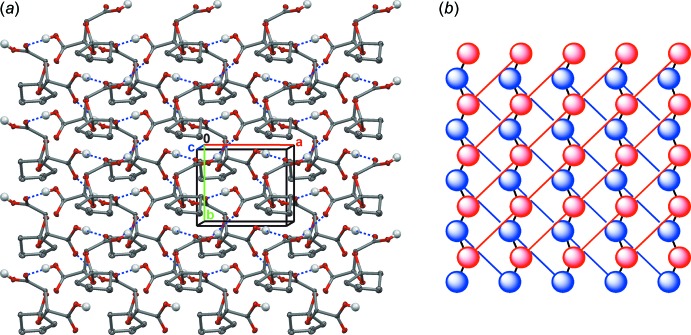
(*a*) The packing of (I) parallel to (001). Two inter­acting mol­ecular layers are shown. Only the H atoms involved in hydrogen bonding (blue dashed lines) have been included. Displacement ellipsoids are drawn at the 50% probability level. (*b*) The simplified structure of the two-dimensional double-layered framework. Mol­ecules (circles) and hydrogen bonds (solid lines) within the same layers are shown in the same colour (blue or red). Hydrogen bonds between two layers are shown as solid black lines.

The mol­ecules of both structures are packed in two-dimensional frameworks by four –CO—OH⋯O=C(OH)– hydrogen bonds between neighboring carboxyl groups (Tables 3 ▸ and 4 ▸). The packing diagrams for (I) (Figs. 4 ▸, 5 ▸ *a*) are nearly identical to those of (II) (not shown). The mol­ecules form double layers parallel to the *ab* plane and sterically shielded from other layers by the cyclo­alkane fragments (Fig. 4 ▸). Hydrogen-bonded chains within the same layer are formed *via* two inter­actions involving the O1—H1 and O3 atoms of each mol­ecule. These chains are inter­connected into a two-dimensional hydrogen-bonded double-layered framework parallel to (001) by the O4—H4 and O2 atoms. The complicated structure of the two-dimensional double-layered framework is shown in Fig. 5 ▸ *a*, but it can best be visualized in the simplified scheme in Fig. 5 ▸ *b*. It might be noted that some weak C—H⋯O inter­molecular inter­actions are also present (see the supporting information).

## Database survey   

Twenty crystal structures of tartaric acid ester derivatives possessing the 1,3-dioxolane cycle, *R*′*R*′′C_3_H_2_O_2_(COO*R*)_2_, are known to date [Cambridge Structural Database (CSD) Version 5.39, latest update Feb 2018; Groom *et al.*, 2016 ▸]. There are 10 crystal structures of esters bearing one substit­uent *R*′ (*R*′′ = H) at the 2-position of the 1,3-dioxolane fragment (acetals): CSD refcodes DAZJET (Lee *et al.*, 1999 ▸), LACREM, LACRUC (Roush *et al.*, 1992 ▸), LEPHAR, LEPHEV (Eissmann *et al.*, 2012 ▸), OLEGAN (Karisalmi *et al.*, 2003 ▸), WEGXOW (Belokon’ *et al.*, 2005 ▸), XEYSEA (Jiang *et al.*, 2007 ▸), YAXHIQ (Lv *et al.*, 2012 ▸) and YIVGUF (Barrett *et al.*, 1995 ▸). The crystal structures of esters with two substituents *R*′ and *R*′′ (ketals) are represented by GAGHAY, GUHGUL (Pelphrey *et al.*, 2004 ▸), KEMRID (Wink & Dewan, 1990 ▸), MIWDIF (Ates & Curran, 2001 ▸), NAFWEW (Mikołajczyk *et al.*, 1996 ▸), QOTVUQ (Maezaki *et al.*, 2000 ▸), VICXOU/VICXOU10 (Giordano *et al.*, 1990 ▸; Ianelli *et al.*, 1992 ▸), VIHVAL (Linker *et al.*, 2013 ▸) and VUCHAC, VUCHEG (Ianelli *et al.*, 1992 ▸). The crystal structures of 14 related amide derivatives *R*′*R*′′C_3_H_2_O_2_(CON*R*_2_)_2_ are also known (see the CSD and also Eissmann *et al.*, 2012 ▸ and references therein). However, established crystal structures of related acids, *R*′*R*′′C_3_H_2_O_2_(COOH)_2_, are limited to only one structure with *R*′ = –C_6_H_4_-4-COOH and *R*′′ = H (LEPHIZ; Eissmann *et al.*, 2012 ▸). This fact can be explained by some subtle problems with the individual isolation of pure acid samples because of the facile hydrolysis of the 1,3-dioxolane fragment during their preparation. Therefore, the synthesis and especially the crystallization of *R*′*R*′′C_3_H_2_O_2_(COOH)_2_ acids is a challenging task.

## Synthesis and crystallization   

### General experimental remarks   

(+)-Diethyl l-tartrate [Sigma–Aldrich, >99%, found [α]_D_^297K^ = +12° (acetone, 20.5mg ml^−1^); lit. data [α]_D_^293K^ = +10° (ethanol, 53 mg ml^−1^), see Černý, 1977 ▸] was used as purchased. ^1^H and ^13^C{^1^H} NMR spectra were recorded with Bruker AM-300 and Bruker DRX-500 spectrometers in CDCl_3_ (Cambridge Isotope Laboratories, Inc., 99.8% ^2^H) and in acetone-*d*_6_ (Sigma–Aldrich, 99.9 atom % ^2^H).

### Synthesis of (2*R*,3*R*)-diethyl 1,4-dioxa­spiro­[4.5]decane-2,3-di­carboxyl­ate   

A 1000 ml round-bottomed flask equipped with a reflux condenser and a Dean–Stark trap was charged with diethyl l-tartrate (85.56 ml, 500 mmol), cyclo­hexa­none (51.82 ml, 500 mmol), toluene (600 ml) and *p*-toluene­sulfonic acid monohydrate (2.80 g, 147 mmol). The mixture was refluxed for 62 h. The resulting dark-brown mixture was washed with a saturated aqueous solution of NaHCO_3_ (2 × 100ml) and with water (2 × 100ml). The organic layer was dried over anhydrous Na_2_SO_4_. The solvent was removed on a rotary evaporator. The obtained dark-brown oil was distilled under reduced pressure (388–391 K, 250 Pa). The yield of the colourless liquid was 84% (120.25 g, 420 mmol). η_D_^293K^ = 1.4625, [α]_D_^297K^ = −28.7 (acetone, 20.5 mg ml^−1^) [Lit. data η_D_^293K^ = 1.4605, [α]_D_^293K^ = −35.57 (Tsuzuki, 1937 ▸)]. ^1^H NMR (CDCl_3_) δ: 1.12 (*t*, 6H, C***H**_3_*—CH_2_–O), 1.30–1.45 (*m*, 10H, –C_5_ ***H**_10_*–), 4.10 (*quartet*, 4H, CH_3_—C***H**_2_*—O), 4.55 (*s*, 2H, C***H***).

### Synthesis of (2*R*,3*R*)-diethyl 1,4-dioxa­spiro­[4.4]nonane-2,3-di­carboxyl­ate   

The synthesis of (2*R*,3*R*)-diethyl 1,4-dioxa­spiro­[4.4]nonane-2,3-di­carboxyl­ate was carried out analogously to that of (2*R*,3*R*)-diethyl 1,4-dioxa­spiro­[4.5]decane-2,3-di­carboxyl­ate, starting from 85.47 ml (500 mmol) of diethyl l-tartrate, 44.23 ml (500 mmol) of cyclo­penta­none, 600 ml of toluene and 2.80 g (14.7 mmol) of *p*-toluene­sulfonic acid monohydrate. The yield of the colourless liquid after vacuum distillation (383–385 K, 265 Pa) was 78% (106.08 g, 390 mmol). ^1^H NMR (CDCl_3_) δ: 1.17 (*t*, 6H, C***H**_3_*—CH_2_—O), 1.51–1.63 (*m*, 4H, –C_4_ ***H**_8_*–), 1.64–1.77 (*m*, 2H, –C_4_ ***H**_8_*–), 1.77–1.91 (*m*, 2H, –C_4_ ***H**_8_*–), 4.12 (*quartet*, 4H, CH_3_—C***H**_2_*—O), 4.57 (*s*, 2H, C***H***).

### Synthesis and crystallization of (2*R*,3*R*)-1,4-dioxa­spiro[4.5]decane-2,3-di­carb­oxy­lic acid, (II)   

A 100 ml round-bottomed flask was charged with 2.130 g (7.52 mmol) of (2*R*,3*R*)-diethyl 1,4-dioxa­spiro­[4.5]decane-2,3-di­carboxyl­ate, 22.5 ml of THF, 22.5 ml of methanol and 22.5 ml of 2 *M* aqueous solution of LiOH. The reaction mixture was stirred for 6 h. It was then washed with diethyl ether (3 × 20 ml). The aqueous solution was acidified with a 2 *M* solution of HCl to pH ≃ 1 at 273 K. The formed acid was extracted with ethyl acetate (3 × 20 ml). The organic layer was dried over Na_2_SO_4_. The solution was removed on a rotary evaporator. The yield of the resulting white powder was 72% (1.250 g, 5.43 mmol). M.p. = 413K, [α]_D_^297K^ = −27.3 (acetone, 20.5 mg ml^−1^) [Lit. data [α]_D_^20^ = −24.0, ethanol, 304 mg ml^−1^ (Innis & Lamaty, 1977 ▸)]. ^1^H NMR (acetone-*d*_6_) δ: 1.36–1.44 (*m*, 2H, —C_5_ ***H**_10_*—), 1.53-1.74 (*m*, 8H, –C_5_ ***H_10_***–), 4.82 (*s*, 2H, C***H***), 7.0 (*br.s*, 2H, –COO***H***). ^13^C{1H} NMR (acetone-*d*_6_) δ: 24.6, 25.6, 36.8, 77.7, 114.7, 171.7. Crystals of (II) were grown from an ethyl acetate/hexane (1:1 *v*/*v*) mixture.

### Synthesis and crystallization of (2*R*,3*R*)-1,4-dioxa­spiro[4.4]nonane-2,3-di­carb­oxy­lic acid, (I)   

The synthesis of (I) was carried out analogously to that of (II), starting from 2.723 g (10 mmol) of (2*R*,3*R*)-diethyl 1,4-dioxa­spiro­[4.4]nonane-2,3-di­carboxyl­ate, 22.5 ml of THF, 22.5 ml of methanol and 22.5 ml of 2 *M* aqueous solution of LiOH. The yield of the resulting white powder was 50% (1.081 g, 5 mmol). ^1^H NMR (acetone-*d*_6_) δ: 1.59–1.72 (*m*, 4H, –C_4_ ***H**_8_*–), 1.74–1.87 (*m*, 2H, –C_4_ ***H**_8_*–), 1.90–2.02 (*m*, 2H, –C_4_ ***H**_8_*–), 4.78 (*s*, 2H, C***H***), 7.5 (*br.s*, 2H, -COO***H***). ^13^C{1H} NMR (acetone-*d*_6_) δ: 24.0, 37.4, 77.9, 123.6, 171.4. Crystals of (I) were grown from an ethyl acetate/hexane (1:1 *v*/*v*) mixture.

## Refinement   

Crystal data, data collection and structure refinement details are summarized in Table 5 ▸. The positions of all non-H and the hy­droxy H atoms were found from the electron difference density maps. These atoms were refined with individual anisotropic (non-H) or isotropic (hy­droxy H) displacement parameters. The positions of the other H atoms were also found from the difference map but they were positioned geometrically (C—H distance = 0.99 Å for methyl­ene, 1.00 Å for tertiary hydrogen atoms) and refined as riding atoms with *U*_iso_(H) = 1.2*U*_eq_(C). Reflection (001) in (II) was affected by the beam stop, and was therefore omitted from the refinement.

## Supplementary Material

Crystal structure: contains datablock(s) I, II, global. DOI: 10.1107/S2056989018009593/eb2009sup1.cif

Structure factors: contains datablock(s) I. DOI: 10.1107/S2056989018009593/eb2009Isup2.hkl

Structure factors: contains datablock(s) II. DOI: 10.1107/S2056989018009593/eb2009IIsup3.hkl

Supporting information file. DOI: 10.1107/S2056989018009593/eb2009Isup4.cml

Supporting information file. DOI: 10.1107/S2056989018009593/eb2009IIsup5.cml

CCDC references: 1853435, 1853434

Additional supporting information:  crystallographic information; 3D view; checkCIF report

## Figures and Tables

**Figure 3:**
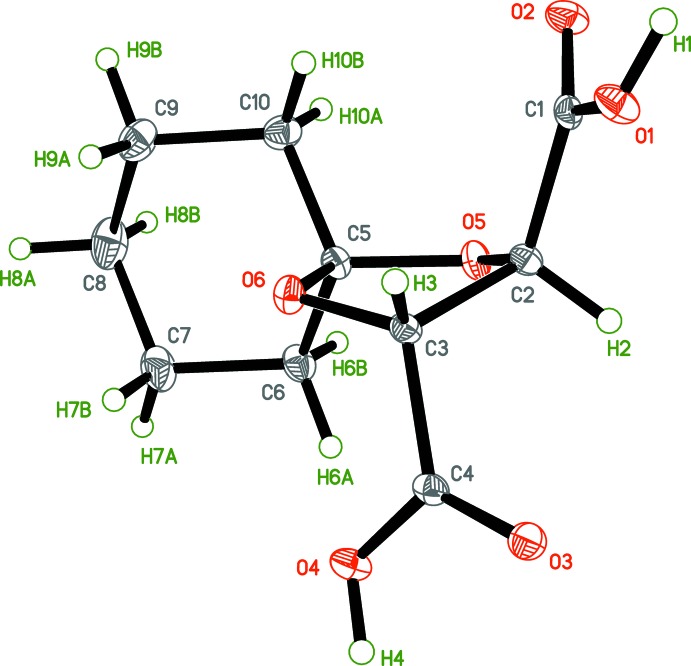
The structure of (2*R*,3*R*)-1,4-dioxa­spiro­[4.5]decane-2,3-di­carb­oxy­lic acid, (II). Displacement ellipsoids are drawn at the 50% probability level.

**Table 1:** Selected bond lengths (Å) for (I)

O1—C1	1.325 (3)	C1—C2	1.521 (4)
O2—C1	1.208 (3)	C2—C3	1.541 (4)
O3—C4	1.222 (3)	C3—C4	1.519 (4)
O4—C4	1.314 (3)	C5—C9	1.529 (4)
O5—C2	1.409 (3)	C5—C6	1.539 (4)
O5—C5	1.443 (3)	C6—C7	1.533 (4)
O6—C3	1.409 (3)	C7—C8	1.529 (4)
O6—C5	1.439 (3)	C8—C9	1.522 (4)

**Table 2:** Selected bond lengths (Å) for (II)

O1—C1	1.322 (2)	C2—C3	1.541 (2)
O2—C1	1.208 (2)	C3—C4	1.532 (2)
O3—C4	1.2229 (19)	C5—C6	1.519 (2)
O4—C4	1.3135 (18)	C5—C10	1.525 (2)
O5—C2	1.4107 (18)	C6—C7	1.533 (2)
O5—C5	1.4398 (18)	C7—C8	1.526 (3)
O6—C3	1.4135 (17)	C8—C9	1.532 (2)
O6—C5	1.441 (2)	C9—C10	1.538 (2)
C1—C2	1.529 (2)		

**Table 3:** Hydrogen-bond geometry (Å, °) for (I)

*D*—H⋯*A*	*D*—H	H⋯*A*	*D*⋯*A*	*D*—H⋯*A*
O1—H1⋯O3^i^	0.78 (4)	1.87 (4)	2.620 (3)	159 (4)
O4—H4⋯O2^ii^	0.84 (4)	1.92 (4)	2.723 (3)	159 (3)

Symmetry codes: (i) 
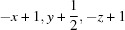
; (ii) 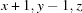.

**Table 4:** Hydrogen-bond geometry (Å, °) for (II)

*D*—H⋯*A*	*D*—H	H⋯*A*	*D*⋯*A*	*D*—H⋯*A*
O1—H1⋯O3^i^	0.84 (3)	1.80 (3)	2.6230 (16)	164 (3)
O4—H4⋯O2^ii^	0.85 (3)	1.88 (3)	2.7116 (16)	164 (2)

Symmetry codes: (i) 
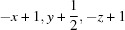
; (ii) 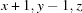.

**Table 5:** Experimental details

	(I)	(II)
Crystal data		
Chemical formula	C_9_H_12_O_6_	C_10_H_14_O_6_
*M*_r_	216.19	230.21
Crystal system, space group	Monoclinic, *P*2_1_	Monoclinic, *P*2_1_
Temperature (K)	100	100
*a*, *b*, *c* (Å)	6.2930 (8), 5.3712 (7), 14.0916 (17)	6.4272 (8), 5.2976 (6), 15.5678 (19)
β (°)	92.885 (2)	94.469 (2)
*V* (Å^3^)	475.71 (10)	528.45 (11)
*Z*	2	2
Radiation type	Mo *K*α	Mo *K*α
μ (mm^−1^)	0.13	0.12
Crystal size (mm)	0.21 × 0.07 × 0.03	0.39 × 0.15 × 0.05

Data collection		
Diffractometer	Bruker SMART APEXII	Bruker SMART APEXII
Absorption correction	Multi-scan (*SADABS*; Bruker, 2008 ▸)	Multi-scan (*SADABS*; Bruker, 2008 ▸)
*T*_min_, *T*_max_	0.827, 0.996	0.917, 0.995
No. of measured, independent and observed [*I* > 2σ(*I*)] reflections	4142, 2455, 1914	4329, 2612, 2503
*R*_int_	0.030	0.015
(sin θ/λ)_max_ (Å^−1^)	0.682	0.682

Refinement		
*R*[*F* ^2^ > 2σ(*F* ^2^)], *wR*(*F* ^2^), *S*	0.043, 0.083, 1.06	0.028, 0.072, 1.05
No. of reflections	2455	2612
No. of parameters	144	153
No. of restraints	1	1
H-atom treatment	H atoms treated by a mixture of independent and constrained refinement	H atoms treated by a mixture of independent and constrained refinement
Δρ_max_, Δρ_min_ (e Å^−3^)	0.29, −0.24	0.30, −0.20

Computer programs: *APEX2* and *SAINT* (Bruker, 2008 ▸), *SHELXS97* and *SHELXTL* (Sheldrick, 2008 ▸), *SHELXL2017* (Sheldrick, 2015 ▸), *Mercury* (Macrae *et al.*, 2008 ▸) and *publCIF* (Westrip, 2010 ▸).

## supplementary crystallographic information

### (2R,3R)-1,4-Dioxaspiro[4.4]nonane-2,3-dicarboxylic acid (I) Crystal data

**Table:**

C_9_H_12_O_6_	*F*(000) = 228
*M**_r_* = 216.19	*D*_x_ = 1.509 Mg m^−^^3^
Monoclinic, *P*2_1_	Mo *K*α radiation, λ = 0.71073 Å
*a* = 6.2930 (8) Å	Cell parameters from 621 reflections
*b* = 5.3712 (7) Å	θ = 3–29°
*c* = 14.0916 (17) Å	µ = 0.13 mm^−^^1^
β = 92.885 (2)°	*T* = 100 K
*V* = 475.71 (10) Å^3^	Needle, colourless
*Z* = 2	0.21 × 0.07 × 0.03 mm

### (2R,3R)-1,4-Dioxaspiro[4.4]nonane-2,3-dicarboxylic acid (I) Data collection

**Table:**

Bruker SMART APEXII diffractometer	2455 independent reflections
Radiation source: fine-focus sealed tube	1914 reflections with *I* > 2σ(*I*)
Graphite monochromator	*R*_int_ = 0.030
ω scans	θ_max_ = 29.0°, θ_min_ = 2.9°
Absorption correction: multi-scan (SADABS; Bruker, 2008)	*h* = −8→8
*T*_min_ = 0.827, *T*_max_ = 0.996	*k* = −7→7
4142 measured reflections	*l* = −14→19

### (2R,3R)-1,4-Dioxaspiro[4.4]nonane-2,3-dicarboxylic acid (I) Refinement

**Table:**

Refinement on *F*^2^	Primary atom site location: structure-invariant direct methods
Least-squares matrix: full	Secondary atom site location: difference Fourier map
*R*[*F*^2^ > 2σ(*F*^2^)] = 0.043	Hydrogen site location: mixed
*wR*(*F*^2^) = 0.083	H atoms treated by a mixture of independent and constrained refinement
*S* = 1.06	*w* = 1/[σ^2^(*F*_o_^2^) + (0.031*P*)^2^] where *P* = (*F*_o_^2^ + 2*F*_c_^2^)/3
2455 reflections	(Δ/σ)_max_ < 0.001
144 parameters	Δρ_max_ = 0.29 e Å^−^^3^
1 restraint	Δρ_min_ = −0.24 e Å^−^^3^

### (2R,3R)-1,4-Dioxaspiro[4.4]nonane-2,3-dicarboxylic acid (I) Special details

**Table:**

Geometry. All esds (except the esd in the dihedral angle between two l.s. planes) are estimated using the full covariance matrix. The cell esds are taken into account individually in the estimation of esds in distances, angles and torsion angles; correlations between esds in cell parameters are only used when they are defined by crystal symmetry. An approximate (isotropic) treatment of cell esds is used for estimating esds involving l.s. planes.
Refinement. Refinement of F^2^ against ALL reflections. The weighted R-factor wR and goodness of fit S are based on F^2^, conventional R-factors R are based on F, with F set to zero for negative F^2^. The threshold expression of F^2^ > 2sigma(F^2^) is used only for calculating R-factors(gt) etc. and is not relevant to the choice of reflections for refinement. R-factors based on F^2^ are statistically about twice as large as those based on F, and R- factors based on ALL data will be even larger.

### (2R,3R)-1,4-Dioxaspiro[4.4]nonane-2,3-dicarboxylic acid (I) Fractional atomic coordinates and isotropic or equivalent isotropic displacement parameters (Å^2^)

**Table:**

	*x*	*y*	*z*	*U*_iso_*/*U*_eq_	
O1	0.3838 (4)	0.5521 (4)	0.45124 (15)	0.0153 (5)	
H1	0.271 (6)	0.587 (8)	0.468 (3)	0.039 (13)*	
O2	0.3808 (3)	0.8867 (4)	0.35486 (14)	0.0156 (5)	
O3	0.9421 (3)	0.1722 (4)	0.44956 (14)	0.0133 (5)	
O4	1.0688 (3)	0.2205 (4)	0.30548 (14)	0.0141 (5)	
H4	1.173 (6)	0.144 (7)	0.332 (2)	0.026 (10)*	
O5	0.7890 (3)	0.7805 (3)	0.30380 (14)	0.0123 (5)	
O6	0.6993 (3)	0.4106 (4)	0.23156 (13)	0.0127 (4)	
C1	0.4679 (4)	0.7060 (5)	0.38994 (19)	0.0108 (6)	
C2	0.6954 (4)	0.6301 (5)	0.3722 (2)	0.0113 (6)	
H2	0.782988	0.639980	0.433289	0.014*	
C3	0.7155 (4)	0.3667 (5)	0.33022 (19)	0.0103 (6)	
H3	0.591522	0.263933	0.348493	0.012*	
C4	0.9202 (4)	0.2424 (5)	0.3672 (2)	0.0105 (6)	
C5	0.7661 (4)	0.6609 (5)	0.2121 (2)	0.0124 (6)	
C6	0.6025 (5)	0.7952 (6)	0.1454 (2)	0.0174 (7)	
H6A	0.481549	0.683619	0.127887	0.021*	
H6B	0.547171	0.945453	0.176471	0.021*	
C7	0.7213 (5)	0.8670 (7)	0.0571 (2)	0.0276 (8)	
H7A	0.704227	0.737514	0.007304	0.033*	
H7B	0.669165	1.027909	0.030732	0.033*	
C8	0.9535 (5)	0.8873 (7)	0.0939 (2)	0.0220 (7)	
H8A	1.051505	0.869535	0.041494	0.026*	
H8B	0.981059	1.048463	0.126290	0.026*	
C9	0.9776 (4)	0.6711 (6)	0.1634 (2)	0.0161 (6)	
H9A	1.097196	0.700938	0.210352	0.019*	
H9B	1.003536	0.513540	0.129416	0.019*	

### (2R,3R)-1,4-Dioxaspiro[4.4]nonane-2,3-dicarboxylic acid (I) Atomic displacement parameters (Å^2^)

**Table:**

	*U*^11^	*U*^22^	*U*^33^	*U*^12^	*U*^13^	*U*^23^
O1	0.0145 (11)	0.0154 (11)	0.0167 (13)	0.0014 (9)	0.0066 (9)	0.0018 (10)
O2	0.0148 (10)	0.0150 (10)	0.0172 (12)	0.0050 (9)	0.0011 (8)	0.0010 (10)
O3	0.0147 (10)	0.0140 (10)	0.0112 (11)	0.0015 (8)	0.0007 (8)	0.0017 (9)
O4	0.0120 (10)	0.0154 (11)	0.0153 (11)	0.0031 (8)	0.0034 (8)	0.0025 (9)
O5	0.0164 (10)	0.0109 (10)	0.0100 (11)	−0.0014 (8)	0.0030 (8)	−0.0012 (8)
O6	0.0178 (10)	0.0102 (9)	0.0099 (11)	−0.0003 (8)	−0.0003 (8)	−0.0005 (9)
C1	0.0146 (13)	0.0093 (12)	0.0085 (14)	−0.0004 (11)	−0.0002 (11)	−0.0040 (12)
C2	0.0136 (14)	0.0087 (13)	0.0117 (16)	0.0012 (10)	0.0015 (11)	−0.0005 (11)
C3	0.0127 (13)	0.0083 (12)	0.0103 (15)	−0.0005 (10)	0.0021 (11)	0.0001 (12)
C4	0.0100 (13)	0.0063 (13)	0.0151 (16)	−0.0005 (10)	0.0001 (11)	−0.0031 (12)
C5	0.0171 (14)	0.0110 (13)	0.0091 (15)	0.0011 (11)	0.0019 (11)	−0.0020 (12)
C6	0.0183 (15)	0.0182 (14)	0.0155 (17)	0.0050 (12)	−0.0003 (13)	−0.0006 (13)
C7	0.0310 (18)	0.035 (2)	0.0167 (18)	0.0068 (16)	0.0021 (14)	0.0092 (16)
C8	0.0258 (16)	0.0225 (16)	0.0185 (18)	0.0003 (14)	0.0089 (13)	0.0071 (15)
C9	0.0164 (14)	0.0163 (15)	0.0160 (17)	0.0014 (12)	0.0047 (12)	0.0012 (14)

### (2R,3R)-1,4-Dioxaspiro[4.4]nonane-2,3-dicarboxylic acid (I) Geometric parameters (Å, º)

**Table:**

O1—C1	1.325 (3)	C3—H3	1.0000
O1—H1	0.78 (4)	C5—C9	1.529 (4)
O2—C1	1.208 (3)	C5—C6	1.539 (4)
O3—C4	1.222 (3)	C6—C7	1.533 (4)
O4—C4	1.314 (3)	C6—H6A	0.9900
O4—H4	0.84 (4)	C6—H6B	0.9900
O5—C2	1.409 (3)	C7—C8	1.529 (4)
O5—C5	1.443 (3)	C7—H7A	0.9900
O6—C3	1.409 (3)	C7—H7B	0.9900
O6—C5	1.439 (3)	C8—C9	1.522 (4)
C1—C2	1.521 (4)	C8—H8A	0.9900
C2—C3	1.541 (4)	C8—H8B	0.9900
C2—H2	1.0000	C9—H9A	0.9900
C3—C4	1.519 (4)	C9—H9B	0.9900
			
C1—O1—H1	117 (3)	O5—C5—C6	111.9 (2)
C4—O4—H4	108 (2)	C9—C5—C6	106.2 (2)
C2—O5—C5	109.3 (2)	C7—C6—C5	106.0 (2)
C3—O6—C5	109.7 (2)	C7—C6—H6A	110.5
O2—C1—O1	125.4 (2)	C5—C6—H6A	110.5
O2—C1—C2	124.0 (2)	C7—C6—H6B	110.5
O1—C1—C2	110.5 (2)	C5—C6—H6B	110.5
O5—C2—C1	112.8 (2)	H6A—C6—H6B	108.7
O5—C2—C3	102.6 (2)	C8—C7—C6	103.9 (3)
C1—C2—C3	113.9 (2)	C8—C7—H7A	111.0
O5—C2—H2	109.1	C6—C7—H7A	111.0
C1—C2—H2	109.1	C8—C7—H7B	111.0
C3—C2—H2	109.1	C6—C7—H7B	111.0
O6—C3—C4	115.5 (2)	H7A—C7—H7B	109.0
O6—C3—C2	102.8 (2)	C9—C8—C7	103.1 (3)
C4—C3—C2	110.9 (2)	C9—C8—H8A	111.1
O6—C3—H3	109.1	C7—C8—H8A	111.1
C4—C3—H3	109.1	C9—C8—H8B	111.1
C2—C3—H3	109.1	C7—C8—H8B	111.1
O3—C4—O4	123.4 (2)	H8A—C8—H8B	109.1
O3—C4—C3	121.0 (2)	C8—C9—C5	104.8 (2)
O4—C4—C3	115.6 (2)	C8—C9—H9A	110.8
O6—C5—O5	105.2 (2)	C5—C9—H9A	110.8
O6—C5—C9	112.9 (2)	C8—C9—H9B	110.8
O5—C5—C9	109.6 (2)	C5—C9—H9B	110.8
O6—C5—C6	111.2 (2)	H9A—C9—H9B	108.9
			
C5—O5—C2—C1	−95.7 (2)	C3—O6—C5—O5	−9.5 (3)
C5—O5—C2—C3	27.4 (3)	C3—O6—C5—C9	110.0 (2)
O2—C1—C2—O5	−5.2 (4)	C3—O6—C5—C6	−130.8 (2)
O1—C1—C2—O5	177.0 (2)	C2—O5—C5—O6	−12.6 (3)
O2—C1—C2—C3	−121.7 (3)	C2—O5—C5—C9	−134.3 (2)
O1—C1—C2—C3	60.5 (3)	C2—O5—C5—C6	108.2 (2)
C5—O6—C3—C4	−95.4 (3)	O6—C5—C6—C7	−120.1 (3)
C5—O6—C3—C2	25.5 (3)	O5—C5—C6—C7	122.6 (3)
O5—C2—C3—O6	−32.0 (2)	C9—C5—C6—C7	3.0 (3)
C1—C2—C3—O6	90.3 (3)	C5—C6—C7—C8	−26.1 (3)
O5—C2—C3—C4	92.0 (3)	C6—C7—C8—C9	39.3 (3)
C1—C2—C3—C4	−145.7 (2)	C7—C8—C9—C5	−37.6 (3)
O6—C3—C4—O3	−172.3 (2)	O6—C5—C9—C8	143.4 (3)
C2—C3—C4—O3	71.3 (3)	O5—C5—C9—C8	−99.7 (3)
O6—C3—C4—O4	8.6 (3)	C6—C5—C9—C8	21.4 (3)
C2—C3—C4—O4	−107.8 (3)		

### (2R,3R)-1,4-Dioxaspiro[4.4]nonane-2,3-dicarboxylic acid (I) Hydrogen-bond geometry (Å, º)

**Table:**

*D*—H···*A*	*D*—H	H···*A*	*D*···*A*	*D*—H···*A*
O1—H1···O3^i^	0.78 (4)	1.87 (4)	2.620 (3)	159 (4)
O4—H4···O2^ii^	0.84 (4)	1.92 (4)	2.723 (3)	159 (3)
C2—H2···O3^iii^	1.00	2.34	3.315 (4)	166
C3—H3···O2^iv^	1.00	2.43	3.358 (3)	155

Symmetry codes: (i) −*x*+1, *y*+1/2, −*z*+1; (ii) *x*+1, *y*−1, *z*; (iii) −*x*+2, *y*+1/2, −*z*+1; (iv) *x*, *y*−1, *z*.

### (2R,3R)-1,4-Dioxaspiro[4.5]decane-2,3-dicarboxylic acid (II) Crystal data

**Table:**

C_10_H_14_O_6_	*D*_x_ = 1.447 Mg m^−^^3^
*M**_r_* = 230.21	Melting point: 413(1) K
Monoclinic, *P*2_1_	Mo *K*α radiation, λ = 0.71073 Å
*a* = 6.4272 (8) Å	Cell parameters from 361 reflections
*b* = 5.2976 (6) Å	θ = 3–29°
*c* = 15.5678 (19) Å	µ = 0.12 mm^−^^1^
β = 94.469 (2)°	*T* = 100 K
*V* = 528.45 (11) Å^3^	Needle, colourless
*Z* = 2	0.39 × 0.15 × 0.05 mm
*F*(000) = 244	

### (2R,3R)-1,4-Dioxaspiro[4.5]decane-2,3-dicarboxylic acid (II) Data collection

**Table:**

Bruker SMART APEXII diffractometer	2612 independent reflections
Radiation source: fine-focus sealed tube	2503 reflections with *I* > 2σ(*I*)
Graphite monochromator	*R*_int_ = 0.015
ω scans	θ_max_ = 29.0°, θ_min_ = 2.6°
Absorption correction: multi-scan (SADABS; Bruker, 2008)	*h* = −8→8
*T*_min_ = 0.917, *T*_max_ = 0.995	*k* = −7→5
4329 measured reflections	*l* = −21→20

### (2R,3R)-1,4-Dioxaspiro[4.5]decane-2,3-dicarboxylic acid (II) Refinement

**Table:**

Refinement on *F*^2^	Primary atom site location: structure-invariant direct methods
Least-squares matrix: full	Secondary atom site location: difference Fourier map
*R*[*F*^2^ > 2σ(*F*^2^)] = 0.028	Hydrogen site location: mixed
*wR*(*F*^2^) = 0.072	H atoms treated by a mixture of independent and constrained refinement
*S* = 1.05	*w* = 1/[σ^2^(*F*_o_^2^) + (0.0411*P*)^2^ + 0.0783*P*] where *P* = (*F*_o_^2^ + 2*F*_c_^2^)/3
2612 reflections	(Δ/σ)_max_ < 0.001
153 parameters	Δρ_max_ = 0.30 e Å^−^^3^
1 restraint	Δρ_min_ = −0.20 e Å^−^^3^

### (2R,3R)-1,4-Dioxaspiro[4.5]decane-2,3-dicarboxylic acid (II) Special details

**Table:**

Geometry. All esds (except the esd in the dihedral angle between two l.s. planes) are estimated using the full covariance matrix. The cell esds are taken into account individually in the estimation of esds in distances, angles and torsion angles; correlations between esds in cell parameters are only used when they are defined by crystal symmetry. An approximate (isotropic) treatment of cell esds is used for estimating esds involving l.s. planes.
Refinement. Refinement of F^2^ against ALL reflections. The weighted R-factor wR and goodness of fit S are based on F^2^, conventional R-factors R are based on F, with F set to zero for negative F^2^. The threshold expression of F^2^ > 2sigma(F^2^) is used only for calculating R-factors(gt) etc. and is not relevant to the choice of reflections for refinement. R-factors based on F^2^ are statistically about twice as large as those based on F, and R- factors based on ALL data will be even larger.

### (2R,3R)-1,4-Dioxaspiro[4.5]decane-2,3-dicarboxylic acid (II) Fractional atomic coordinates and isotropic or equivalent isotropic displacement parameters (Å^2^)

**Table:**

	*x*	*y*	*z*	*U*_iso_*/*U*_eq_	
O1	0.38484 (18)	0.5840 (2)	0.45476 (7)	0.0142 (2)	
H1	0.275 (4)	0.638 (6)	0.4745 (17)	0.046 (8)*	
O2	0.37180 (17)	0.9308 (2)	0.37124 (7)	0.0150 (2)	
O3	0.92950 (17)	0.2135 (2)	0.45684 (7)	0.0124 (2)	
O4	1.05961 (17)	0.2630 (2)	0.32878 (7)	0.0134 (2)	
H4	1.158 (4)	0.171 (5)	0.3519 (15)	0.027 (6)*	
O5	0.76550 (18)	0.8366 (2)	0.32166 (7)	0.0118 (2)	
O6	0.70176 (17)	0.4547 (2)	0.25475 (7)	0.0116 (2)	
C1	0.4607 (2)	0.7451 (3)	0.40041 (9)	0.0104 (3)	
C2	0.6846 (2)	0.6758 (3)	0.38313 (9)	0.0099 (3)	
H2	0.774689	0.688258	0.438295	0.012*	
C3	0.7100 (2)	0.4108 (3)	0.34451 (9)	0.0099 (3)	
H3	0.588324	0.303877	0.357764	0.012*	
C4	0.9117 (2)	0.2852 (3)	0.38196 (10)	0.0103 (3)	
C5	0.7505 (2)	0.7152 (3)	0.23867 (9)	0.0109 (3)	
C6	0.9603 (2)	0.7413 (3)	0.20088 (9)	0.0145 (3)	
H6A	1.069512	0.658928	0.239569	0.017*	
H6B	0.996255	0.922393	0.196647	0.017*	
C7	0.9553 (3)	0.6204 (4)	0.11126 (11)	0.0208 (4)	
H7A	1.090749	0.648573	0.086579	0.025*	
H7B	0.934204	0.435992	0.116324	0.025*	
C8	0.7800 (3)	0.7324 (4)	0.05127 (10)	0.0233 (4)	
H8A	0.775479	0.646187	−0.005267	0.028*	
H8B	0.807902	0.913725	0.041928	0.028*	
C9	0.5691 (3)	0.7025 (4)	0.08968 (10)	0.0191 (3)	
H9A	0.535628	0.520808	0.094378	0.023*	
H9B	0.458492	0.782178	0.050960	0.023*	
C10	0.5734 (2)	0.8254 (3)	0.17929 (10)	0.0151 (3)	
H10A	0.593000	1.009889	0.173941	0.018*	
H10B	0.438602	0.795642	0.204323	0.018*	

### (2R,3R)-1,4-Dioxaspiro[4.5]decane-2,3-dicarboxylic acid (II) Atomic displacement parameters (Å^2^)

**Table:**

	*U*^11^	*U*^22^	*U*^33^	*U*^12^	*U*^13^	*U*^23^
O1	0.0135 (5)	0.0139 (6)	0.0160 (5)	0.0025 (5)	0.0065 (4)	0.0026 (5)
O2	0.0139 (5)	0.0142 (6)	0.0172 (5)	0.0044 (4)	0.0029 (4)	0.0027 (5)
O3	0.0127 (5)	0.0125 (6)	0.0122 (5)	0.0009 (4)	0.0014 (4)	0.0004 (4)
O4	0.0115 (5)	0.0133 (6)	0.0157 (5)	0.0032 (4)	0.0036 (4)	0.0017 (5)
O5	0.0156 (5)	0.0092 (5)	0.0111 (5)	−0.0026 (4)	0.0036 (4)	−0.0012 (4)
O6	0.0156 (5)	0.0087 (5)	0.0104 (5)	−0.0011 (4)	0.0005 (4)	0.0002 (4)
C1	0.0114 (6)	0.0102 (7)	0.0097 (6)	−0.0006 (5)	0.0010 (5)	−0.0031 (6)
C2	0.0101 (6)	0.0087 (7)	0.0110 (6)	0.0007 (5)	0.0020 (5)	0.0006 (5)
C3	0.0097 (6)	0.0090 (7)	0.0111 (6)	0.0015 (5)	0.0011 (5)	−0.0007 (5)
C4	0.0099 (6)	0.0066 (7)	0.0145 (7)	−0.0003 (5)	0.0008 (5)	−0.0011 (5)
C5	0.0125 (6)	0.0095 (7)	0.0108 (6)	0.0003 (5)	0.0020 (5)	−0.0007 (6)
C6	0.0141 (7)	0.0158 (8)	0.0142 (6)	−0.0018 (6)	0.0048 (5)	−0.0016 (6)
C7	0.0203 (8)	0.0265 (10)	0.0165 (7)	−0.0028 (7)	0.0079 (6)	−0.0053 (7)
C8	0.0288 (9)	0.0291 (10)	0.0126 (7)	−0.0068 (8)	0.0054 (6)	−0.0003 (7)
C9	0.0208 (8)	0.0239 (10)	0.0122 (7)	−0.0016 (7)	−0.0008 (6)	0.0028 (7)
C10	0.0149 (7)	0.0158 (8)	0.0145 (7)	0.0015 (6)	0.0000 (5)	0.0029 (6)

### (2R,3R)-1,4-Dioxaspiro[4.5]decane-2,3-dicarboxylic acid (II) Geometric parameters (Å, º)

**Table:**

O1—C1	1.322 (2)	C5—C10	1.525 (2)
O1—H1	0.84 (3)	C6—C7	1.533 (2)
O2—C1	1.208 (2)	C6—H6A	0.9900
O3—C4	1.2229 (19)	C6—H6B	0.9900
O4—C4	1.3135 (18)	C7—C8	1.526 (3)
O4—H4	0.85 (3)	C7—H7A	0.9900
O5—C2	1.4107 (18)	C7—H7B	0.9900
O5—C5	1.4398 (18)	C8—C9	1.532 (2)
O6—C3	1.4135 (17)	C8—H8A	0.9900
O6—C5	1.441 (2)	C8—H8B	0.9900
C1—C2	1.529 (2)	C9—C10	1.538 (2)
C2—C3	1.541 (2)	C9—H9A	0.9900
C2—H2	1.0000	C9—H9B	0.9900
C3—C4	1.532 (2)	C10—H10A	0.9900
C3—H3	1.0000	C10—H10B	0.9900
C5—C6	1.519 (2)		
			
C1—O1—H1	112 (2)	C5—C6—H6A	109.4
C4—O4—H4	109.4 (15)	C7—C6—H6A	109.4
C2—O5—C5	109.65 (12)	C5—C6—H6B	109.4
C3—O6—C5	109.70 (12)	C7—C6—H6B	109.4
O2—C1—O1	125.41 (14)	H6A—C6—H6B	108.0
O2—C1—C2	123.59 (14)	C8—C7—C6	110.88 (15)
O1—C1—C2	110.89 (13)	C8—C7—H7A	109.5
O5—C2—C1	112.10 (12)	C6—C7—H7A	109.5
O5—C2—C3	103.28 (11)	C8—C7—H7B	109.5
C1—C2—C3	114.66 (12)	C6—C7—H7B	109.5
O5—C2—H2	108.9	H7A—C7—H7B	108.1
C1—C2—H2	108.9	C7—C8—C9	110.75 (14)
C3—C2—H2	108.9	C7—C8—H8A	109.5
O6—C3—C4	114.38 (12)	C9—C8—H8A	109.5
O6—C3—C2	103.84 (12)	C7—C8—H8B	109.5
C4—C3—C2	111.08 (12)	C9—C8—H8B	109.5
O6—C3—H3	109.1	H8A—C8—H8B	108.1
C4—C3—H3	109.1	C8—C9—C10	110.87 (14)
C2—C3—H3	109.1	C8—C9—H9A	109.5
O3—C4—O4	123.68 (14)	C10—C9—H9A	109.5
O3—C4—C3	120.74 (14)	C8—C9—H9B	109.5
O4—C4—C3	115.58 (13)	C10—C9—H9B	109.5
O5—C5—O6	105.80 (12)	H9A—C9—H9B	108.1
O5—C5—C6	107.89 (12)	C5—C10—C9	110.35 (14)
O6—C5—C6	111.46 (13)	C5—C10—H10A	109.6
O5—C5—C10	111.57 (13)	C9—C10—H10A	109.6
O6—C5—C10	108.05 (13)	C5—C10—H10B	109.6
C6—C5—C10	111.92 (13)	C9—C10—H10B	109.6
C5—C6—C7	110.97 (13)	H10A—C10—H10B	108.1
			
C5—O5—C2—C1	−99.25 (14)	C2—O5—C5—O6	−12.62 (15)
C5—O5—C2—C3	24.70 (14)	C2—O5—C5—C6	−132.02 (13)
O2—C1—C2—O5	−6.9 (2)	C2—O5—C5—C10	104.65 (14)
O1—C1—C2—O5	176.76 (12)	C3—O6—C5—O5	−6.39 (15)
O2—C1—C2—C3	−124.22 (16)	C3—O6—C5—C6	110.62 (13)
O1—C1—C2—C3	59.43 (16)	C3—O6—C5—C10	−126.00 (13)
C5—O6—C3—C4	−100.36 (14)	O5—C5—C6—C7	−178.91 (14)
C5—O6—C3—C2	20.87 (14)	O6—C5—C6—C7	65.34 (17)
O5—C2—C3—O6	−27.60 (14)	C10—C5—C6—C7	−55.80 (19)
C1—C2—C3—O6	94.65 (14)	C5—C6—C7—C8	55.80 (19)
O5—C2—C3—C4	95.81 (13)	C6—C7—C8—C9	−56.5 (2)
C1—C2—C3—C4	−141.94 (13)	C7—C8—C9—C10	56.9 (2)
O6—C3—C4—O3	−172.39 (14)	O5—C5—C10—C9	176.83 (13)
C2—C3—C4—O3	70.47 (18)	O6—C5—C10—C9	−67.27 (16)
O6—C3—C4—O4	7.99 (19)	C6—C5—C10—C9	55.82 (18)
C2—C3—C4—O4	−109.15 (15)	C8—C9—C10—C5	−56.08 (19)

### (2R,3R)-1,4-Dioxaspiro[4.5]decane-2,3-dicarboxylic acid (II) Hydrogen-bond geometry (Å, º)

**Table:**

*D*—H···*A*	*D*—H	H···*A*	*D*···*A*	*D*—H···*A*
O1—H1···O3^i^	0.84 (3)	1.80 (3)	2.6230 (16)	164 (3)
O4—H4···O2^ii^	0.85 (3)	1.88 (3)	2.7116 (16)	164 (2)
C2—H2···O3^iii^	1.00	2.41	3.3818 (19)	164
C3—H3···O2^iv^	1.00	2.44	3.392 (2)	160
C6—H6*A*···O4	0.99	2.52	3.255 (2)	131

Symmetry codes: (i) −*x*+1, *y*+1/2, −*z*+1; (ii) *x*+1, *y*−1, *z*; (iii) −*x*+2, *y*+1/2, −*z*+1; (iv) *x*, *y*−1, *z*.
